# Pancreatic β Cell Mass Death

**DOI:** 10.3389/fphar.2016.00083

**Published:** 2016-04-06

**Authors:** Husnia I. Marrif, Salma I. Al-Sunousi

**Affiliations:** ^1^Department of Pharmacology, Faculty of Medicine, University of BenghaziBenghazi, Libya; ^2^Department of Histology and Anatomy, Faculty of Medicine, University of BenghaziBenghazi, Libya

**Keywords:** β cells, apoptosis, insulin, protein folding, stem cells, diabetes, autophagy

## Abstract

Type two diabetes (T2D) is a challenging metabolic disorder for which a cure has not yet been found. Its etiology is associated with several phenomena, including significant loss of insulin-producing, beta cell (β cell) mass via progressive programmed cell death and disrupted cellular autophagy. In diabetes, the etiology of β cell death and the role of mitochondria are complex and involve several layers of mechanisms. Understanding the dynamics of those mechanisms could permit researchers to develop an intervention for the progressive loss of β cells. Currently, diabetes research has shifted toward rejuvenation and plasticity technology and away from the simplified approach of hormonal compensation. Diabetes research is currently challenged by questions such as how to enhance cell survival, decrease apoptosis and replenish β cell mass in diabetic patients. In this review, we discuss evidence that β cell development and mass formation are guided by specific signaling systems, particularly hormones, transcription factors, and growth factors, all of which could be manipulated to enhance mass growth. There is also strong evidence that β cells are dynamically active cells, which, under specific conditions such as obesity, can increase in size and subsequently increase insulin secretion. In certain cases of aggressive or advanced forms of T2D, β cells become markedly impaired, and the only alternatives for maintaining glucose homeostasis are through partial or complete cell grafting (the Edmonton protocol). In these cases, the harvesting of an enriched population of viable β cells is required for transplantation. This task necessitates a deep understanding of the pharmacological agents that affect β cell survival, mass, and function. The aim of this review is to initiate discussion about the important signals in pancreatic β cell development and mass formation and to highlight the process by which cell death occurs in diabetes. This review also examines the attempts that have been made to recover or increase cell mass in diabetic patients by using various pharmacological agents.

## Introduction

Diabetes mellitus (DM) is a cluster of chronic metabolic disorders triggered by insulin deficiency. The incidence and prevalence of DM are high and are steadily increasing worldwide (Koloverou et al., 2014; Samuel-Hodge et al., 2014; Anjana et al., 2015; Luo et al., 2015; Pati et al., 2015; Srinivasan and Florez, 2015; Tancredi et al., 2015). The American Diabetes Association has classified diabetes into four categories: type one diabetes (T1D), type two diabetes (T2D), Gestational diabetes mellitus (GDM), and other specific types of diabetes (American Diabetes Association, 2015). In T1D, there is a complete failure of the pancreas to produce insulin. In contrast, in T2D, there is a fundamental change in insulin secretion and in insulin receptor dynamics.

In T2D research, a plethora of studies have sought to rescue pancreatic β cells and maintain insulin secretion (Butler et al., 2007). Insulin is secreted by the pancreas, an organ that hosts various cell lineages, many of which are involved in endocrine or exocrine functions. The present review, however, focuses on the β cells of the islets of Langerhans, which are responsible for insulin secretion.

Current research in diabetes reveals that a significant reduction in pancreatic β cell populations is the leading cause of the decrease in insulin production in T2D. A significant decrease in β cell mass was first reported when pancreatic samples from autopsies of diabetic patients were compared with samples from non-diabetic subjects (Butler et al., 2003). Available studies also suggest that the decline in β cell mass precedes the onset of diabetes by almost a decade. Furthermore, studies show that at the time of diagnosis, diabetic patients seem to have already lost ~50% of their pancreatic β cells. This loss is a slow process that ends with the individual experiencing full-blown diabetes. The mechanism by which β cell mass decreases is still under investigation; however, the available evidence suggests that hyperglycemia induces apoptotic cell death, which may be the key pathological mechanism of diabetes.

Events affecting the β cell population are classified into two groups. The first group is composed of factors influencing β cell lineage expression, neogenesis, and maturation (molecular events). The second group includes factors causing mature β cell injury and death (cellular events). Unfortunately, the biological processes are more complex than these classifications imply; for instance, metabolic factors that cause direct cell injury or death can also affect the neogenesis and lineage commitment of β cells. This review focuses on the factors that affect β cell mass.

## Overview of pancreas development and functions

Since the discovery of insulin deficiency in diabetic individuals, endocrine researchers have made it a priority to acquire knowledge about the development of the pancreas, its cellular endocrinology, and its pathophysiology an utmost priority.

The development of the pancreas commences with the budding of the ventral and dorsal region from the embryogenic layer of the endoderm. Most of our collective knowledge of cellular pancreatic growth comes from studies on rodents; with the exception of a few deviations, rodents exhibit pancreatic development quite similar to that in humans. The budding of the pancreas in a mouse starts at day E9.5. The ductal system is formed before E13; at that point, cells begin to differentiate (Gao et al., 2008). One of the most important signals in this stage is initiated by the homeobox transcription factor (Pdx1) in the pancreas and duodenum. The pancreas then proceeds to the second stage of cellular maturity or lineage commitment, with cells terminally differentiating into either endocrine or exocrine cells. This phase is guided by a specific set of transcription factors and growth signals (Gittes, 2009).

## Cellular development

Much like that of any other organ, the embryonic development of the pancreas establishes the setting for proper cell maturation and function. Cells influenced by environmental and genetic factors can travel along any of the following pathways: generation, proliferation, differentiation, and possibly full lineage commitment, quiescence state (dedifferentiation), or mutation. The above possibilities are governed by the presence of endogenous and/or exogenous signals, which can include hormones, growth factors, neurotransmitters, intermediate metabolites, or transcription factors (Bonner-Weir et al., 2010). Numerous transcription factors act as key signals for the proliferation and terminal differentiation of pancreatic cells.

## The mature pancreas

Upon inspection of the mature organ, a gross view of the pancreas shows a simplified ductal system surrounding the head, body, and tail of the pancreas, with scattered islets of cells at different sites. The cellular structure of the pancreas, however, is far more complex. This level of detail exhibits a complex “community” of cells with variable shapes and functions. Pancreatic products, whether hormonal or non-hormonal, are secreted by distinct populations of cells, each at a specific location and with a specific function (Table 1). Ultimately, cells in the pancreas can be classified as either endocrine or exocrine cells, depending on their function.

**Table 1:** **Pancreatic cell populations and functions**.

**Cell**	**Secretion**	**Target**	**Function**
**ENDOCRINE CELLS OF THE PANCREASES**			
A α (alpha)	Glucagon	Liver	Increases plasma glucose
	Glucagon-like peptides (GLP-1) and (GLP-2)	Pancreas	Increases insulin secretion and sensitivity. It decreases glucagon secretion
B (Beta)	Insulin	All sites	Decreases plasma glucose
	Amylin or Islet Amyloid Polypeptide (IAPP)	Pancreas	Decreases Pancreatic enzymes
		Gut	Decreases gastric emptying
D δ (Delta)	Somatostatin	Pancreas	Inhibits insulin and glucagon secretion
F PP	Pancreatic polypeptide	Pancreas	Regulates secretion
(ε) Epsilon	Ghrelin (AG). Unacylated Ghrelin (UAG)	Pancreas	Inhibits insulin release and increases appetite
	Obestatin	Pancreas	Increases insulin release and decreases appetite
**EXOCRINE CELLS OF THE PANCREAS**			
Acinar cells	Alpha-amylase, proteases and lipases	Gut	Promotes digestion
	Islet neogenesis associated protein (peptide of 175 amino acids)	Pancreas	Induces β cell neogenesis
Duct Cells	Forming ductal structure, secrete mucus, bicarbonate	Gut	Promotes digestion and regenerative process

## Pancreatic exocrine cells

### Acinar cells

The exocrine functions of the pancreas are carried out by exocrine cells, also known as acinar cells. The name acinar, or *acini* as a group, refers to the aggregation of these cells into clusters. The main physiological function of acinar cells is to secrete pancreatic digestive enzymes (e.g., alpha-amylase, proteases, and lipases). The mixture is then emptied into the duodenum via the ductal system. Regarding the contribution of these cells to pancreatic cell development and lineage commitment, acinar cell function goes well beyond only secretion. For example, these cells are involved in regulating the neogenesis of islet cells (Table 1).

### Duct cells

The ductal structure of the pancreas is also formed by epithelial cells derived from the pancreatic primordia. These duct cells are connected in a chain-like structure to form convoluted tubing throughout the pancreas, and their main physiological function is to secrete mucus and bicarbonate. Current research suggests that the function of duct cells exceeds that of their exocrine duties, much like the function of acinar cells. Given their important role in the regenerative process in the pancreas, duct cells are discussed in greater detail at a later point in this review.

## Pancreatic endocrine cells

### Islets of langerhans: development, function, and manipulation

The pancreas contains exocrine acinar and ductal cells, and endocrine cells that form the islets of Langerhans. The islet cells can be classified into five distinct glandular cell types: alpha (α), beta (β), delta (δ), epsilon (ε), and F cells (Table 1). In humans, the pancreas contains an estimated one million islet cells (Bonner-Weir et al., 2010), and the islets occupy ~1–1.5% of the organ's volume. The exocrine cells occupy ~95% of the pancreas in adult humans and rodents (Hara et al., 2007).

Rodents are the most widely used experimental model for studying pancreatic cells. However, there are a few notable differences between the islet cells of rodents and humans. For instance, during the developmental stages of humans and rodents, β cells are found in the core of the islets and are surrounded by δ and α cells (Steiner et al., 2010). In rodents, this basic structure continues to exist in adults. In adult humans, however, β cells are found scattered throughout the pancreas, although there is a high density in the anterior portion of the pancreas head (Yesil and Lammert, 2008; Steiner et al., 2010).

There is also evidence that human and rodent islets are equipped with different glucose sensor systems. Research shows that human islets use Glut-1 and Glut-3, whereas Glut-2 is the main glucose transporter in rodents (McCulloch et al., 2011; Rorsman and Braun, 2013). Different transporters have different affinities (*Km*-value) for glucose; this difference is crucial, as it eventually influences insulin release.

Another important difference is growth rate; *in vitro*, human β cells grow at a much slower rate than do those of rodents (Szkudelski, 2001).

Estimates of β cell content are essential for diabetes research, as these estimates can be used in cell rescue therapy and grafting. Current studies show variable estimates of β cell content; therefore, it is essential to consider the differences between the experimental models used (e.g., species, age, and technical procedures) and the measures used to describe β cell content, such as volume or fraction. It has been reported that the adult human pancreas contains nearly one million islets (Matveyenko and Butler, 2006; Rorsman and Braun, 2013). In addition, a recent study using a combination of light and electron microscopy reported that 73.6 ± 1.7% of human islet cells were β cells (Pisania et al., 2010).

### β cell proliferation vs. differentiation

**Figure 1:**
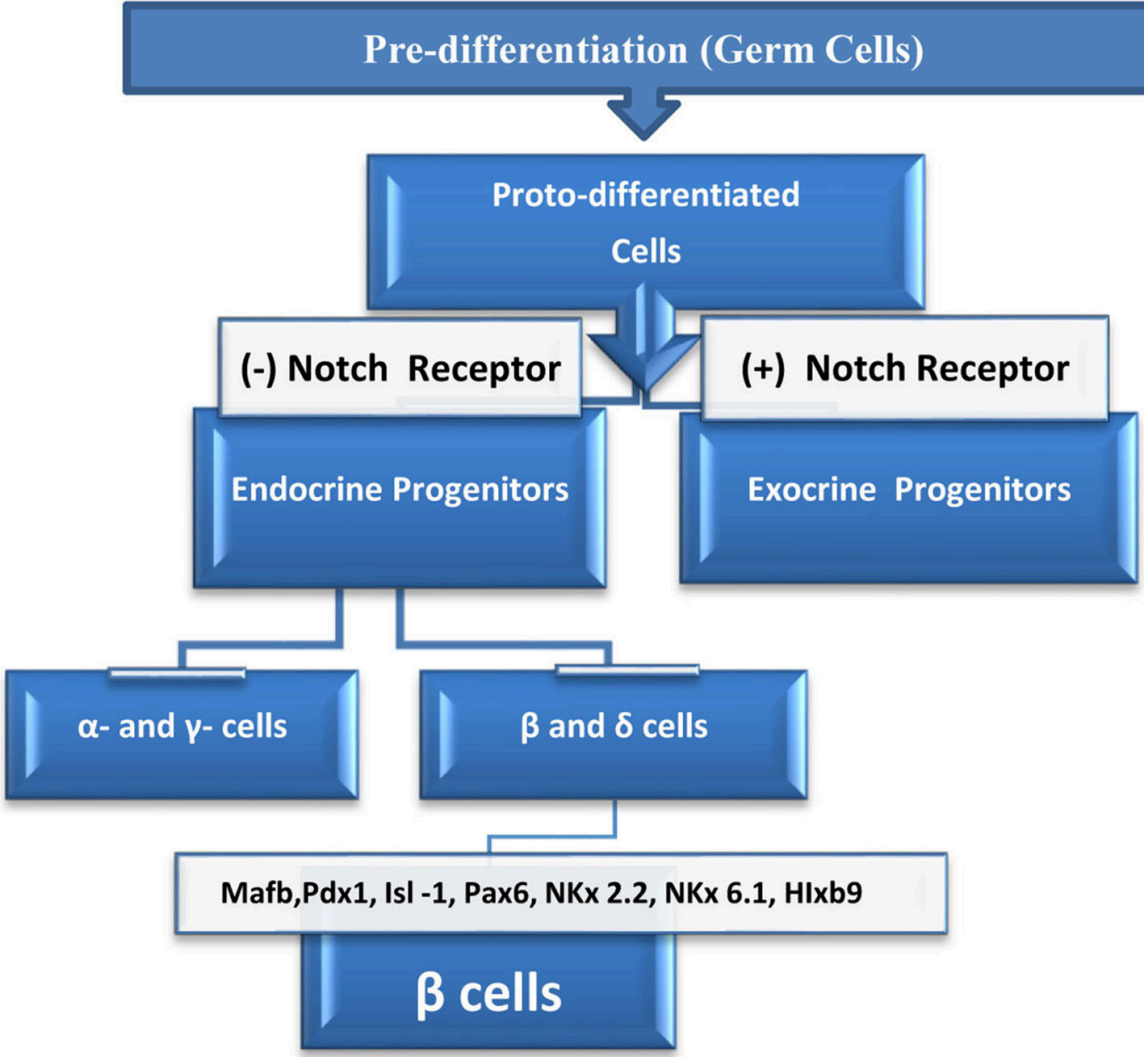
**Life cycle of β cells starts at the embryonic germinal stage where pre-differentiated cells changed into proto-differentiated stage under a specific signal**. Thereafter, cells express Notch protein which bind to Notch receptor and activate downstream genes, and cells become exocrine progenitors. Cells which lack Notch protein expression, proceeds to form subset of endocrine progenitor cells which continue to differentiate into alpha (α), epsilon (ε) or to beta (β), and delta (δ) cells. In the presence of the transcriptional signals above, beta (β), and delta (δ) cells continue differentiation to become β cells which eventually mature to insulin producing cells.

Rodent studies suggest that the fetal stage is the most critical period in pancreatic β cell mass formation. At this point, islet β cells start to multiply in response to growth factors, hormones and, probably, critical signals from the autonomic nervous system (Kiba, 2004). During lineage formation or differentiation, cells undergo three stages of development—pre-differentiation, proto-differentiation, and differentiation (Figure 1)—and each stage is regulated by specific signals. Islets contain five cell lineages, all of which originate from endodermal progenitor cells, and the development of these lineages is guided by either specific signals or common signals.

Some growth signals have a well-defined role in differentiation and proliferation (Table 2); in most cases, however, the exact functions of growth signals are still obscure (Estefanía et al., 2012). In addition to domestic cycle cell regulators, some local signals have been found to affect β cell differentiation and phenotypes (Table 2). One key determining factor in β cell fate is the Notch signaling pathway. It has been suggested that the Notch 1 receptor, which is expressed in many of the lineages found in islets, can halt exocrine and endocrine cell differentiation, with endocrine cell differentiation being essential for cells to acquire insulin-producing and insulin-sensing properties.

**Table 2:** **Signals that affect pancreatic development and growth**.

**Signal mediator**	**Location and note**	**References**
Homeobox HB9 (Hlxb9)	Dorsal region of pancreas; endocrine and exocrine cells; involved in β cell differentiation	Li et al., 1999.
Pancreas transcription factor 1 complex (Ptf1a)	Ventral region and pancreas; endocrine and exocrine cell differentiation	Schaffer et al., 2010
bHLH transcription factor 1 (Hes1)	Transcriptional repressor; keeps progenitor cells undifferentiated	Jensen et al., 2000
bHLH transcription factor Mist1	Ventral region and pancreas; endocrine and exocrine cell phenotyping	Pin et al., 2001
Epidermal growth factor (EGF) Hepatocyte growth factor (HGF) Platelet-derived growth factor (PDGF) Transforming growth factor alpha (TGF-alpha) and insulin	Stimulates progenitor cell proliferation via receptor tyrosine kinases	Yesil and Lammert, 2008
Insulin-like growth factor (IGF-1 and 2)	Stimulates progenitor cells	Bouwens and Rooman, 2005
Homeoprotein Isl-1 (Islet-1)	Stimulates the development of the dorsal pancreatic bud; β cell differentiation and proliferation	Du et al., 2009
Neurogenin 3 (Ngn-3)	bHLH family; stimulates endocrine cell differentiation	Gradwoh et al., 2000; Song et al., 2004; Gunasekaran et al., 2012
Homeobox Protein Nkx-6.1 (Nkx6.1) and Homeobox Protein Nkx-2.2 (Nkx2.2)	β cell differentiation	Oster et al., 1998
Paired box gene 4 (Pax-4)	Transcriptional repressor for alpha cells and enhancer for β cells in early development	Smith et al., 1999
Paired box gene 6 (Pax-6)	β cell differentiation and function	Hart et al., 2013
Pancreatic and duodenal homeobox 1 (Pdx-1)/ insulin promoter factor-1	β cell differentiation and function	Hui and Perfetti, 2002
Growth hormone (GH)	β cell proliferation	Nielsen et al., 1989
Prolactin (PRL)	β cell differentiation and morphogenesis	Auffret et al., 2013
Placental lactogen (PL)	β cell proliferation and phenotype	Fleenor et al., 2000
Parathyroid hormone-related protein (PTHrP)	β cell differentiation and proliferation	Vasavada et al., 1996
Gastrin	Neogenesis and transdifferentiation	Rooman et al., 2002
Glucose-dependent insulinotropic polypeptide (GIP)	Reduce β cell death (anti-apoptotic) and modulate function	McIntosh et al., 2009
Glucagon-like peptide 1 (GLP-1)	β cell proliferation	Buteau et al., 2003
Cyclin D1 and D2	β cell proliferation	Kushner et al., 2005
Cdk4	β cell proliferation	Martín et al., 2003

## Factors affecting β cell development

### Notch signaling pathway

The Notch family of receptors are transmembrane transcription factors. The activated intracellular domain of Notch family members interacts with the DNA-binding protein RBP-Jk, thereby activating the expression of the basic negative helix–loop–helix (bHLH) HES genes. This, in turn, represses the expression of downstream target genes (Ehebauer et al., 2006). The expression of Notch proteins in differentiated cells inhibits the phenotyping of certain cells in their vicinity via a process called lateral inhibition. Cells that do not respond to the Notch signal undergo differentiation (Murtaugh et al., 2003).

Notch signaling is the only factor for which there is sufficient evidence to show that it can decide the fate of pancreatic cells. In the absence of the Notch signal, β cells commence differentiation, developing the properties of mature β cells, such as the expression of Glut-2, Glut-1, and glucokinase, as well as the secretion of insulin. When the Notch signal is expressed at a normal level, cells tend to differentiate into exocrine cells. A general outline of the factors that affect cell differentiation is illustrated in Figure 1. Lineage studies have identified other factors that are associated with β cell lineage development, such as Mafb, Isl-1, Pax6, and Pax-4, HIxb9, NKx2.2, and Nkx6.1. As noted above, some factors are common regulators of the islet's lineages; however, the quantities expressed in each lineage are critical for specific lineage's execution.

## Progenitor cells

Discussion about the role of neogenesis began when studies reported that the ductal cells surrounding the islets can generate β cells and likely other types of cells (Lipsett and Finegood, 2002; Song et al., 2004; Gunasekaran et al., 2012). The ability of β cells to proliferate from mature cells or other cell lineages initiated intense scientific debate and curiosity.

**Figure 2:**
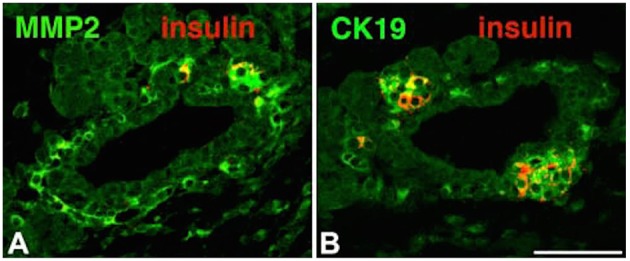
**Immunohistochemistry of islet markers for morphogenesis including; matrix metalloproteinase-2 (MMP-2), cytokeratin 19 (CK19) and insulin**. MMP-2 and Ck19 appeared in **A** and **B** sections (green). Insulin positive cells (Red) budding from the ducts appeared in section close to ducts **(B)**. Adapted from Aye et al. (2010). Copyright © 2010 by SAGE Publications. Reprinted with permission from SAGE Publications.

In short, neogenesis, or the regeneration of endocrine β cells, occurs within a cluster of epithelial exocrine duct cells. The process commences when a subset of endocrine progenitor cells, buried within the ductal cells, starts to express a lineage marker known as Neurogenin 3 (Ngn3) by a process known as “budding.” Cells then separate from the ductal chain to form the characteristic aggregates of insulin-secreting islets (Figure 2). Further studies have reported that the progenitor cells can give rise to all lineages of pancreatic endocrine cells, including alpha (α), beta (β), and epsilon (ε) cells (Cabrera et al., 2006).

## Neurogenin-3

The protein Neurogenin was first identified in both mice and Xenopus (Ma et al., 1996). The family includes Neurogenin-1 (Ngn1), Neurogenin-2 (Ngn2), and Neurogenin-3 (Ngn3). Whereas, Ngn1 and Ngn 2 were identified in the central nervous system, Ngn-3 is localized in pancreatic endocrine cells. Neurogenins are transcription factors that control the expression of large sets of downstream genes that are directly involved in neogenesis (Rukstalis and Habener, 2009).

Neurogenin belongs to the family of basic helix-loop-helix (bHLH) transcription factors; several members of this family are factors that influence differentiation and sub-lineage formation (Sommer et al., 1996). Neurogenin is one of the earliest regulators and determinants of neurogenesis in vertebrates. Pancreatic Notch proteins control Ngn3 expression, and the Ngn3 signal controls several downstream transcription factors, including Nkx2.2, Pax-4, and NeuroD. All of the signals noted above are critical genes for β cell differentiation and phenotyping (Figure 1). Lineage tracing and commitment studies demonstrate that the expression of Ngn3 in progenitor cells is transient and that the amount expressed is critical for cell lineage commitment (Rukstalis and Habener, 2009).

## Cell lineage tracing

Cell lineage tracing is a technique designed to detect the presence of a particular lineage. One of the most widely used genetic tools is Cre-lox recombination, or site-specific recombinase technology. This technology involves the recombinase enzyme Cre, which recombines a pair of short target sequences, called lox sequences. This system allows the user to insert or delete gene markers, which can then be detected during cell development; it is widely used for tracing pancreatic β cells (Wajchenberg, 2007). New techniques are also available, such as injecting dye into specific cells via viruses (Xiao et al., 2014).

## β cell mass formation

In rodents, the process of β cell mass formation continues at a high rate in the fetal and newborn stages; this mass formation is followed by a significant slowdown in cell proliferation as rodents age, and a similar process occurs in human pancreatic development. Studies in mice using mitotic indexes have estimated that 10–20% of β cell mass is formed by simple cell division (Swenne and Eriksson, 1982). Mitosis comes to a halt when mature cells that secrete insulin are detected in the islet, and this stage represents the terminal differentiation stage of β cells (Murtaugh et al., 2003).

Approximately 80% of β cell mass formed during the fetal stage is thought to result from the differentiation of “undifferentiated” progenitor cells and/or from the conversion of non-endocrine cells into endocrine cells (Bouwens and Rooman, 2005). Studies on animals demonstrate that in adults, β cell mass increases mainly via a β cell lineage replication process rather than by conversion or neogenesis (Dor et al., 2004).

This observation is critical because it limits the search for a source of replenishment of β cells. Apoptosis plays a detrimental role in β cell mass formation. During the neonatal developmental stage, β cells in pigs, humans, and rodents undergo a period of transient apoptosis that ultimately limits the number of β cells that is capable of multiplying at more advanced stages of growth (Bouwens and Pipeleers, 1998). Another factor that can modulate pancreatic β cell mass is, in quantitative terms, the number of bulk progenitor cells in the pancreas that enters the quiescent stage and never differentiates; currently, in the context of diabetes, this process is described as the dedifferentiation of β cells (Ziv et al., 2013).

In summary, humans are born with a limited number of β cells, and those cells preferentially increase their mass through mitosis, if needed. Obesity is one condition that can result in an increase in β cell mass (Kloppel et al., 1985; Plesner et al., 2014). However, the factors and signals that control the epigenetics of β cell growth and death in obesity are still unclear and require further investigation (Tennant et al., 2013).

## Collective cellular growth

The growth of pancreatic cells seems to be part of a collective community process in which communication is essential for organ development and maturation. Animal studies suggest that neighboring cells can influence each other's mass via several mechanisms. For instance, a study by Plesner et al. (2014) reported an intriguing autoimmune destructive process that was triggered by pancreatic α cells neighboring β cells, which ultimately resulted in the reduction of β cell mass and the appearance of hyperglycemia.

Another example is the presence of a small peptide secreted by pancreatic acinar cells, known as “islet neogenesis-associated protein” (INGAP). INGAP is a member of the Reg protein family, which can induce β cell neogenesis and proliferation (Tam et al., 2002). It has been observed that the incubation of the INGAP peptide with human adipose tissue-derived stem cells leads to the differentiation of fat cells into islet-like clusters. Moreover, when these islet-like clusters were implanted into a rat model of T2D, the transplanted tissue improved the glycemic index and diabetic symptoms (Ren et al., 2014).

In a conditional transgenic model in which INGAP was specifically expressed in exocrine pancreas cells, mice showed normalized glycemic indices and exhibited improved responses to hyperglycemia following a diabetogenic dose of streptozotocin.

The protective effect of INGAP might be mediated by different mechanisms, which could include the reduction of oxidative stress as well as INGAP might have pleiotropic properties (Chang et al., 2011). Recently, research on INGAP has generated interest in β cell exogenesis and the development of a mechanism through which insulin secretion can be maintained. There is evidence that INGAP is also effective in clinical settings. Trials have reported that the INGAP peptide improves glycemic index and increases insulin production in patients with T2D (Barbosa et al., 2006; Lipsett et al., 2007; Wang et al., 2010). As this peptide also affects the way in which the body uses insulin, the same beneficial effects were reported in T1D patients. Several new clinical trials to investigate this peptide are currently registered with the US government.

## Transdifferentiation

Transdifferentiation is the technical term for the process of generating β cells from other cell lineages. Scientifically, the concept of converting previously committed lineages to insulin-secreting β cells seems rather daring, and the field is still evolving. Animal models and lineage tracing have provided strong evidence for the transdifferentiation of adult cell lineages, such as duct cells (Bonner-Weir et al., 2008) and pancreatic acinar cells, into β cells (Minami and Seino, 2008). At present, acinar cell research seems to be the most promising for β cell regeneration; however, this should not be confused with research in which stem cells are isolated and genetically altered to become β cells (Ren et al., 2014).

## Yamanaka factors

One of the most intriguing recent discoveries in the field of cell biology is the development of the induced pluripotent stem cell (iPSC) protocol. The original protocol involved the introduction into adult cells of four genes that encode the following transcription factors: KLF4 (Kruppel-like factor 4), Oct-4 (octamer-binding transcription factor 4), SRY (sex determining region Y-box 2), and Myc (c-Myc). This cocktail converts adult cell lineages into pluripotent stem cells (Takahashi and Yamanaka, 2006). The mere concept of transforming mature, committed cells into pluripotent stem cells that can be manipulated to grow into any lineage is bold and innovative. It reflects the unlimited possibilities of cell manipulation and the ability of cells to be transformed into any lineage.

For this discovery, a deserving Shinya Yamanaka shared the Nobel Prize in Physiology or Medicine in 2012. Since the Yamanaka protocol was published, several studies have demonstrated the possibility of manipulating adult cells into insulin-producing cells (Thatava et al., 2013; Shaer et al., 2015). The protocol to produce rich β cell cultures is still being refined and is not without hurdles. For instance, culture contamination and high rates of apoptosis are two of the many challenges (Ziv et al., 2013).

## Mature human β cells

The breakthrough by Yamanaka described above opened the door to research into “pancreatic plasticity” and to transcription factors other than the ones he specified that could manipulate cell neogenesis. Currently, the list of transcription factors includes Foxo1, Zbed6, Pdx1, Ngn3, MafA, Pax4, PPRβ/δ, and Arx, as well as proteins such as Txnip and Sh2b1 (Collombat et al., 2009).

Mature β cell proliferation under simulated physiological condition is highly controversial and many studies reported contradictory results. For instance, it has been observed that when β cells are isolated from young mice (8 weeks old) and are incubated with human growth hormone and liraglutide, a long-acting glucagon-like peptide-1 receptor agonist that is approved by the FDA for T2D treatment, the result is a rich and highly proliferated mass of insulin-secreting cells. Under the same experimental conditions, β cells cultured from adult human donors (ages 16–64) failed to show any proliferation (Parnaud et al., 2008). It seems that adult human cells have a limited capacity for proliferation. Furthermore, genetic and tracing studies suggest that the conversion of cells and the presence of stem cells at the adult stage are limited or non-existent. The only exceptions to this process are the pancreatic acinar cells, which are direct progeny of embryonic multi-potent progenitor cells (Bonner-Weir et al., 2010). Under special circumstances such as obesity, the β cell mass has been shown to have the ability to increase in size. After birth, the proliferation of pancreatic β cells is assumed to be limited; however, a substantial increase in β cell mass, by up to 50%, has been observed in obese subjects (Kloppel et al., 1985).

This unique process is considered to be a compensatory mechanism for hyperglycemia and decreased β cell mass. It may also be evidence for the presence of either a subset of β cells that has the capacity to proliferate or a “magic bullet” factor that forms in response to obesity. Accordingly, questions have arisen regarding the source of the β cell increase and the guiding signals that could lead β cells to proliferate in obese individuals (Ferrannini et al., 2004; Bouwens and Rooman, 2005). Yamanaka's protocol and other similar *in vitro* observations provide opportunities to harvest rich β cell masses that could be used for pancreatic tissue regrowth and transplantation to treat diabetic patients.

## β cells and diabetes

The main function of pancreatic β cells is to sense insulin needs and produce enough hormones to decrease the amount of glucose to its physiological level, regardless of when food was last ingested. The pathophysiology of diabetes commences when the insulin level is inadequate to decrease the blood glucose level and the patient develops hyperglycemia. T1D is defined as autoimmune destruction of pancreatic β cell mass, and its management is centered around insulin replacement, maintaining tissue sensitivity to insulin and controlling body weight.

The pathophysiology of T2D is different from that of T1D and is a combination of a reduction in β cell mass and an increase in insulin resistance. The reduction in β cell mass has been confirmed in animal models of diabetes and obesity. Whether β cell destruction is achieved by chemicals or by genetic manipulation, as in transgenic and knockout models, the endpoint is a reduction in β cell mass and hyperglycemia (Marrif et al., 1995; King, 2012). Studies using animal models have shown that symptoms of glucose intolerance, and eventually hyperglycemia, appear when the density of β cells in the pancreas falls below the threshold value of 10 million per kilogram of body weight (Wang et al., 1996). The most successful animal models used for studying β cell mass injury and generation are the duct ligation models. It has been reported that when 90% of the pancreas is resected, a remarkable hyperglycemia is produced and is followed by a significant regeneration of the pancreas (Bonner-Weir et al., 1983).

The regenerative process observed in this model starts as early as 60 h after the procedure. The ligation model (in which the tail of the pancreas is ligated) leads to a significant decrease in pancreas size (50–60%), which eventually leads to a nearly doubled β cell mass in the tail area; 60% partial-pancreatectomy in mice evokes a similar regenerative process (Peshavaria et al., 2006). This unique restoration process is characterized by an increase in β cell and alpha cell populations and islet clusters, as well as an upregulation of glucose transporter two (GLUT2) in ductal cells. Based on tracing methods, β cell neogenesis is attributed to a significant increase in ductal cell proliferation (Wang et al., 1995). Human clinical cases have also provided evidence that in the event of a tumor or pancreatitis, pancreatectomy can produce a specific regenerative process that restores β mass homeostasis (Schlegel et al., 2000).

Other models can also be used to study β cell mass damage and regeneration, such as the chemical ablation of β cell mass or genetically manipulated models. The latter type of model is more useful for studying β cell regeneration, as the timing of β cell mass ablation can be controlled; examples are the doxycycline-induced expression of diphtheria toxin in a β cell model and the diphtheria toxin receptor-RIP mouse model. There are additional knockout and transgenic models that target specific genes (King, 2012). Considering all of the evidence for β cell mass reduction in type 2 diabetes, the collective challenge now for diabetes research is to find a way to increase β cell mass quantitatively.

## Diabetes induces β cell death

The most critical clinical study of the pathophysiology of diabetics is that of Butler et al. (2003). The authors collected 124 post-mortem human pancreatic samples, including samples from individuals who had been diagnosed with T2D, non-diabetic individuals, individuals with impaired fasting glucose levels, and lean individuals with diabetes. They investigated the following parameters: the frequency of apoptosis and replication, the β cell/islet volume, and new islet formation (Butler et al., 2003).

Their results offered the first glimpse of the human pancreas after the onset of diabetes. Their results are summarized as follows: they found that a significant reduction in β cell volume by ~40–63% (*P* < 0.05) takes places in diabetics and those with glucose intolerance compared with that of non-diabetics.

The study also reported two important observations. First, there is a significant increase (*P* < 0.05) in the frequency of apoptosis, representing a staggering three- to ten-fold increase in apoptotic cell death in diabetic patients. Second, they found evidence of β cell neogenesis or proliferation within the pancreatic ductal system in all the groups that were tested. The study is particularly relevant because it is the largest human study to date that has shed light on the mechanism by which β cell death occurs in diabetes. It is also the first study to provide evidence that the regeneration of β cells in the human pancreas is possible.

There is further evidence supporting the notion that the β cell population is a mass that effectively responds to increases in demand through increases in proliferation. An earlier study of human pancreatic autopsies showed an increase in β cell mass of ~40% in both obese T2D and obese non-diabetic individuals (Kloppel et al., 1985). The increase in β cell mass in obese individuals and diabetic individuals is considered a compensatory mechanism (Kloppel et al., 1985; Butler et al., 2003). Chronic exposure to hyperglycemia causes β cell insults leading to cell death, and the body compensates for the lost cells by increasing the β cell mass. This cycle continues until the ability of the pancreas to generate new cells is exhausted. Current research describes the rate of β cell volume reduction in diabetes as a slow and ongoing process, and it is estimated that β cell death in diabetes probably begins 10–12 years before an individual is diagnosed (Wajchenberg, 2007).

## Sophisticated cell death caused by a simple sugar

Clinical studies have shown that a deviation from glycemic homeostasis, or hyperglycemia, is the main etiology for the development of diabetes and diabetes complications (The Diabetes Control and Complications (DCCT) Trial Research Group, 1993, 1998). Glycemic homeostasis is a collective, harmonized process that involves a network of hormones, peptides, and neurotransmitters, and the most critical physiological function is that of insulin.

In healthy individuals, insulin tightly controls blood glucose within a particular range, postprandial or otherwise. In T1D, insulin is absent from the circulation and has to be replaced. In T2D, there is a shortage of insulin. However, insulin is not the only hormone relevant to glucose homeostasis. For instance, the glucagon hormone that is produced by pancreatic α cells, which increases liver glucose production, is essential for maintaining the glucose level within healthy limits. It has been reported that the manipulation of glucagon by receptor agonists, such as glucagon-like peptide-1 (GLP-1), or by dipeptidyl peptidase-4 (DPP-4) inhibitors can decrease glucagon levels and eventually reduce the degree of hyperglycemia and its associated risks (D'Alessio, 2011; Park et al., 2015). Incretin, or hormone glucose-dependent insulinotropic peptide (GIP), is yet another critical hormone in glucose homeostasis and stimulates glucagon secretion and the eventual state of hyperglycemia.

Another class of drugs that directly reduces hyperglycemia is sodium-glucose cotransporter type 2 (SGLT2) inhibitors. This family includes dapagliflozin, empagliflozin, canagliflozin, and gliflozin. These compounds act by blocking glucose reabsorption by proximal tubules in the kidney. They reportedly improve the glycemic index and contribute to weight loss in animal models of diabetes (Liang et al., 2012) and in diabetic patients (Oguma et al., 2015).

The SGLT2 knockout model has shown that blocking glucose absorption in the kidneys can preserve the β cell population and decrease cell death (Jurczak et al., 2011). There is also evidence that using SGLT2 blockers can preserve β cell mass and function (Hansen et al., 2014). However, the FDA warns of an increase in the incidence of ketoacidosis associated with the use of SGLT2 inhibitors. The long-term use of this drug class requires further study (Kibbey, 2015).

## Hyperglycemia and apoptosis

**Figure 3:**
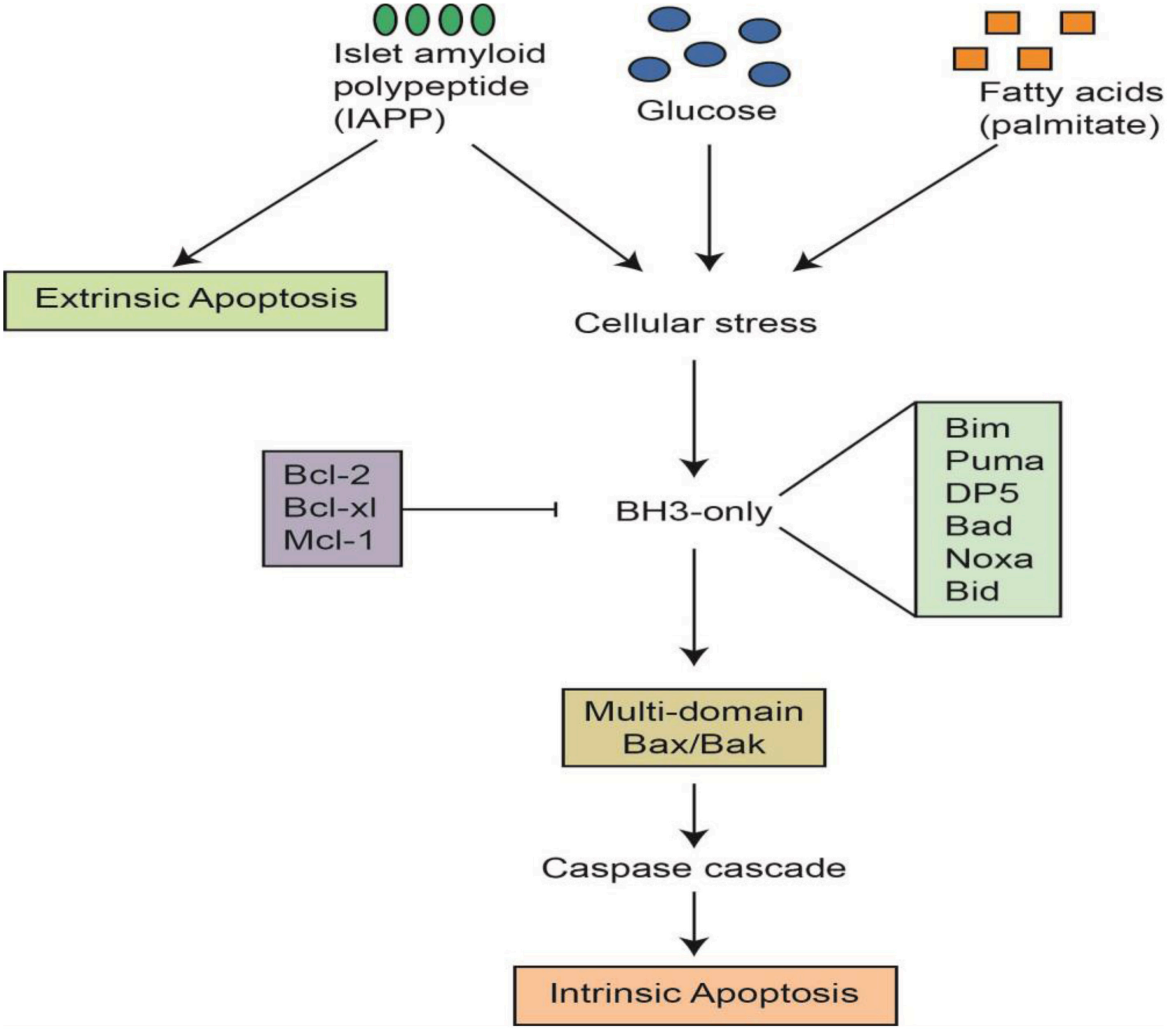
**Apoptotic signals and cascades in pancreatic β cells**. This figure was graciously provided by Helen Thompson and Jibran Wali, Islet Biology Laboratory, St Vincent's Institute, Australia.

In diabetes, β cell death can be mediated by various etiological factors, including the exposure of cells to glucotoxicity (e.g., hyperglycemia and carbohydrate metabolites), lipotoxicity (e.g., triglycerides, LDL, cholesterol, and oxidation products), and pro-inflammatory mediators (e.g., cytokines). Of all the factors noted above, hyperglycemia is the most notable contributor; it is the hallmark of diabetes and the smoking gun (Figure 3). It has been reported that chronic exposure to hyperglycemia alone is capable of initiating apoptotic β cell death in cell cultures and animal models (Elmore, 2007; Marroqui et al., 2015), and this has also been observed in humans through autopsy (Butler et al., 2003).

Although programmed cell death is well studied and the cascades and pathways are well mapped, the pathways by which hyperglycemia activates β cell death in diabetes are not entirely understood. It has been reported that hyperglycemia can produce dose-dependent programmed cell death in β cell cultures. One study has shown that when MIN6N8 (insulinoma) cells are exposed to hyperglycemia (33.3 mmol/l glucose), significant programmed cell death occurs in a time- and dose-dependent fashion when compared with cells incubated with 5.5 mmol/l glucose (Kim et al., 2005).

There is also evidence that chronic hyperglycemia in β cell cultures activates the Bad genes, which are proapoptotic. Bad transcription factors are dual-purpose, in that they simultaneously activate apoptotic genes and inhibit the survival pathways of cells; their action depends on their state of phosphorylation (Datta et al., 2002; Wali et al., 2013).

Mitochondria are directly involved in apoptosis, but the exact signals and events occurring in β cell mitochondria that lead to cell failure are still under investigation. Apoptosis involves multiple mechanisms, including the depolarization of the outer mitochondrial membrane, the opening of the permeability transition pore (PTP) in the inner membrane, the efflux of cytochrome c, the release of Ca^2+^ from the mitochondrial storage site, and the release of a 50-kDa apoptosis-inducing factor (Heiskanen et al., 1999). Moreover, β cell apoptosis can be initiated via different factors, such as signals from surrounding cells (Kikuta et al., 2013; Mcllwain et al., 2013), mitochondrial energy substrates, or metabolites such as inorganic phosphates (Pramanik et al., 2011).

To better understand the etiology of β cell death by apoptosis in diabetes, the mechanisms have been classified, according to etiology, as intrinsic events (via mitochondria), or extrinsic events (via receptors; Figure 3). For instance, apoptosis can be instigated by hyperglycemia, obesity, inflammatory mediators, or beta amyloid peptides (Wali et al., 2013). All of the above factors, alone or in-combination, have the ability to trigger apoptotic cell death, yet hyperglycemia has been shown to be the most likely culprit in β cell apoptosis. T2D is a progressive disease, patients are frequently exposed to hyperglycemia, and each episode triggers more β cell apoptosis.

Clinically, almost all therapeutic agents used for the treatment of T2D manipulate hyperglycemia either directly or indirectly; the question is whether they can also preserve β cell mass. There is evidence that some therapeutic agents used for the treatment of T2D have the ability to reduce β cell apoptosis, aside from other pharmacological properties. For instance, an *in vitro* study has shown that thiazolidinediones are capable of inhibiting β cell apoptosis induced either by a combination of hyperglycemia (25 mM glucose) and 0.5 mM palmitate (Han et al., 2008) or by cytokines (Chou et al., 2007; Li et al., 2010).

## ERS and mitochondrial function

As demonstrated in Figure 3, numerous pathways can mediate β cell death, but most of the mechanisms either finish or start with the mitochondria. Mitochondria oversee energy production through two main processes: the electron transport chain reaction and oxidative phosphorylation (Lin and Beal, 2006). The process of energy production is reportedly concurrent with proton and electron leaks that eventually facilitate the generation of different types of reactive oxygen species (ROS; Cadenas and Davies, 2000; Jastroch et al., 2010; Affourtit et al., 2012). The class of ROS includes the superoxide ion (O${}_{2}^{-}$), the hydroxyl radical (OH^−^), and nitric oxide (NO^−^) as well as the non-radicals hydrogen peroxide (H_2_O_2_), singlet oxygen (O_2_), hypochlorous acid (HOCl), and peroxynitrite (NO${}_{3}^{-}$; Vara and Pula, 2014).

It is believed that at a basic level, ROS can serve as physiological signals (Zorov et al., 2014). However, if the production of ROS exceeds cell capacity, these reactive species can substantially damage cell components, including the mitochondria, via lipid peroxidation, protein oxidation, and DNA mutation (Pacher et al., 2007).

In the pancreas, ROS can further contribute to β cell failure by damaging the apparatus that is essential for insulin synthesis or by depleting the energy required for this process (Lagouge and Larsson, 2013). Animal models of T2D have shown an association of diabetes with the production of ROS (Carlsson et al., 1999; Ihara et al., 1999; King, 2012). *In vitro* β cell culture studies have supported this observation along with other signs of β cell failure, such as a significant depletion of cellular energy and cell death (Fu et al., 2015).

Many clinical studies have shown a pattern of hyperglycemia that is associated with ROS (Tessier et al., 1999; Marra et al., 2002); β cells, in particular, have a low capacity to avoid damage caused by ROS because these cells have the lowest expression of antioxidant enzymes, such as superoxide dismutase (SOD), catalase, and glutathione peroxidase (Lenzen et al., 1996). Furthermore, the overexpression of antioxidant enzymes, such as glutathione peroxidase-1 (GPx-1), in an animal model of diabetes provided protection against hyperglycemia-induced oxidative stress (Harmon et al., 2009). Some animal models and *in vitro* studies have shown that some antioxidants can ameliorate cell damage in diabetes (Kaneto et al., 1999; Koya et al., 2003).

More specifically, by targeting the mitochondria, the antioxidant mitoquinone (MitoQ) exerts a protective effect against glucotoxicity-induced β cell apoptosis (Lim et al., 2011). Mitoquinone is a member of a new therapeutic class of antioxidants that specifically targets the mitochondria; it is a ubiquinone derivative designed to accumulate in high concentrations in the mitochondrial matrix (Smith et al., 2003). Another mitochondria-targeting agent is ruboxistaurin, an orally active protein kinase C beta inhibitor (PKCI) that has been tested in diabetes complication trials (Bansal et al., 2013; Tuttle et al., 2015). Ruboxistaurin is reportedly effective for the prevention of retinopathy (Gogula et al., 2013), renopathy (Alicic and Tuttle, 2014), and peripheral neuropathy (Vinik et al., 2005; Bansal et al., 2013). However, some clinical trials testing ruboxistaurin as well as other non-specific antioxidants, such as vitamin E, vitamin C, coenzyme Q10, alpha lipoic acid, and L-carnitine, have failed to show any beneficial clinical effects (Golbidi et al., 2011).

The pharmacodynamics of PKCIs in clinical trials are not clearly understood (Mochly-Rosen et al., 2012). Ruboxistaurin has not yet been approved by the FDA, and further studies are required to demonstrate its efficacy (Bansal et al., 2013; Sheetz et al., 2013). The argument against PKCI use is based on the fact that kinases are involved in almost all cell functions, and finding a selective PK iso-enzyme inhibitor is a particularly challenging task. The role of protein kinases and their inhibitors in the β cell mass is also not entirely clear. However, evidence suggests that the overexpression of PKC delta increases β cell proliferation via an increase in the phosphorylation of p21. Furthermore, under stressful conditions, PKC delta activates its proapoptotic function and can mediate apoptotic β cell death (Ranta et al., 2011).

### Autophagy

The processes of cell injury and apoptosis are associated with a phenomenon known as autophagy, a process by which cells eliminate damaged organelles via lysosomal degradation and facilitate new cell component turnover. Recently, it has been proposed that the etiology of several diseases, including diabetes, might involve a dysfunction of the autophagy process. Studies of the relation between diabetes and autophagy have shown that when β cells and human islet cells are exposed to high levels of glucose or lipids, the process of autophagy is blocked; subsequently, oxidized, damaged, or mis-folded proteins accumulate in the cells. These sequential events result in signs of β cell stress and failure (Fujimoto et al., 2009; Lee et al., 2012; Mir et al., 2015).

Various signals regulate autophagy. One class of proteins has been reported to be part of the autophagy machinery; mTOR complex 1 (mTORC1) and mTOR complex 2 (mTORC2) proteins. mTOR proteins are kinases that are involved in several cellular processes, including insulin resistance, adipogenesis, angiogenesis, and autophagy (Laplante and Sabatini, 2009). These kinases are thought to be upregulated in certain diseases and involved in dysfunctional autophagy. With respect to pancreatic β cells, it has been reported that when β cells are incubated with rapamycin, the cells undergo autophagy and signs of glucose impairment appear. When β cells are incubated with 3-methyladenine, which inhibits autophagy, cell viability returns to normal (Tanemura et al., 2012). Recent animal studies have shown that the onset of diabetes may be associated with protein misfolding, a compromised autophagy process and endoplasmic reticulum stress (ERS; Quan et al., 2012; Fu et al., 2015; Maiese, 2015).

### Protein misfolding

Endoplasmic reticulum (ER) protein misfolding has drawn considerable attention in recent years as a possible etiology for several chronic diseases, including diabetes, non-alcoholic fatty liver disease, cancer, and Alzheimer's disease. The ER is one of the organelles responsible for storing Ca^2+^ as well as for protein folding, assembly, and biosynthesis. Moreover, in β cells, the ER is the site of insulin biosynthesis (Harding and Ron, 2002). The notion of ER stress in diabetes originated after several studies concluded that during the pre-diabetic period, β cells entered a hyper-active mode of pro-insulin synthesis, which led to an increase in protein misfolding; the mis-folded proteins eventually accumulated at the ER and caused ERS (Kaufman, 2002; Dobson, 2003; Sitia and Braakman, 2003; Thomas et al., 2011; Kim et al., 2012).

In the pre-diabetic stage, two processes occur: insulin resistance and an overexpression of, proinsulin and unfolded protein species in the ER lumen (Ron and Walter, 2007; Fonseca et al., 2011). The accumulation of mis-folded proteins is believed to lead to the ERS of β cells and is associated with an inability to clear mis-folded proteins or dysfunctional autophagy. This process is associated with a substantial generation of ROS, apoptosis and β cell death (Contreras et al., 2003). A stressful event triggers a stress signal, which subsequently activates a downstream network known as the unfolded protein response (UPR; Karaskov et al., 2006; Chambers et al., 2007). Two transmembrane kinases, IREI and PERK, and one transcription factor, ATF6, have been identified within the sensory nexus of the membrane; in the case of stress, these kinases send signals to halt protein translation, increase protein folding, enhance cell autophagy, and initiate cell apoptosis (Papa, 2015).

The latter process can be mediated either through the activation of a CCAAT/enhancer-binding homologous protein (CHOP; Yu et al., 2015) or through the activation of apoptosis signaling kinases (Eitel et al., 2003), a process by which the body recycles damaged proteins (Eitel et al., 2003).

All of the above mechanisms are aspects of cell repair and maintenance (Laybutt et al., 2007). Unraveling the sequence of these events should improve the potential for therapeutic intervention (Cohen and Kelly, 2003). For instance, ERS can be reversed using liraglutide, which prevents ERS-induced apoptosis in β cells (Zhao et al., 2013).

The upstream controlling signals that initiate ERS can include frequent hyperglycemia (Prentki and Nolan, 2006), high levels of free fatty acids and inflammatory cytokines (Cardozo et al., 2005). Mitochondria and the ER are major regulators of intracellular Ca^2+^ levels. Any interference with Ca^2+^ storage or homeostasis can trigger a second sequence of pathological processes that alter normal metabolism and can lead to cell death (Giorgi et al., 2012).

The proper functioning of β cells is reportedly disrupted by abnormal Ca^2+^ levels; for instance, insulin exocytosis from storage granules depends on the Ca^2+^ level, a process that is highly unregulated in diabetes (Fonseca, 2009). Insulin release from β cells can be stimulated by glucose levels and mediated by Ca^2+^ levels. The latter enters cells via voltage-dependent Ca^2+^ channels, which then activate several kinases, and the main insulin release signaling is carried out via Ca^2+^/calmodulin-dependent protein kinase II (CaMKII; Tabuchi et al., 2000). CaMKII activity is tightly correlated with diabetes symptoms, and inhibition of the enzyme complex reportedly produces an impaired glucose tolerance (Dadi et al., 2014); β cells store Ca^2+^ in mitochondria and the ER, and damage to either organelle can greatly alter both Ca^2+^ levels and insulin release (Dixit et al., 2013).

## β cell grafting and pancreas plasticity

At present, there are two branches of diabetes research. The first is directed toward understanding the factors influencing β cell mass maintenance and preservation, which will directly benefit T2D patients by making products that can preserve β cell mass available. This group of products includes those such as amylin agonist pramlintide, GLP-1 receptor agonists, and dipeptidyl peptidase-4 (DPP-4) inhibitors (Godoy-Matos, 2014), and many other products are under development. The second research branch is concentrated on developing an understanding of how to increase β cell mass *in vitro* for the sole purpose of pancreatic transplantation or grafting.

The best example is the Edmonton protocol, in which pancreas islets are transplanted into T1D patients (Shapiro et al., 2000). Many breakthroughs have been made in this particular area of research, such as the transplantation of a bio-artificial pancreas that provides special shielding for the β cell graft against the recipient immune system, which is a technology known as βAir (Street et al., 2004; Barkai et al., 2013). This technique is currently being tested in clinical trials. The original target for pancreas transplantation was T1D; however, animal studies have demonstrated that β cell grafting (partial transplanting) is also a possible avenue of exploration for T2D patients (Close et al., 2005).

## Conclusion

Diabetes is a chronic metabolic disorder that is an epidemic problem. Its pathophysiology revolves around a unique phenomenon of programmed β cell death. The study of both normal β cell mass growth and β cell mass pathophysiology is essential for the design of any intervention. Pancreatic cells influence one another's survival, and their growth is guided by signals that affect cell development and phenotyping.

The adult human pancreas has a limited stock of β cells, which, in diabetess, is ccontinuously exposed to insults, leading to a decrease in mass. To replenish these cells or to replace them by grafting, more research is required to pinpoint the exact mechanism associated with β cell death and the factors affecting cell proliferation. Several pharmacological agents have been shown to restore β cell mass. However, clinically, many of these compounds are associated with side effects. Current evidence directly implicates mitochondria in the β cell death in diabetes. Therefore, understanding the role of mitochondria and mitochondrial agents in preserving the insulin secretion of β cells is critical. Our understanding of the pathophysiology of the β cell death associated with obesity, hyperglycemia or other causes should provide key information for the development of more specific agents for preserving β cell mass.

## Author contributions

SA covered the section: introduction up to the type of cells in pancreas. The rest of the review was written by HM.

### Conflict of interest statement

The authors declare that the research was conducted in the absence of any commercial or financial relationships that could be construed as a potential conflict of interest.
